# Effect of processed sweet lupin (*Lupinus angustifolius*) grain supplementation on growth performance and socioeconomic feasibility of Doyogena sheep in Ethiopia

**DOI:** 10.1002/vms3.883

**Published:** 2022-07-27

**Authors:** Habite Tilaye, Bimrew Asmare, Fentahun Meheret, Melkamu Bezabih, Wamatu Jane

**Affiliations:** ^1^ Department of Animal Sciences Bahir Dar University Bahir Dar Ethiopia; ^2^ International Livestock Research Institute Feeds and forages development program Addis Ababa Ethiopia; ^3^ International Center for Agricultural Research in the Dry Areas Addis Ababa Ethiopia

**Keywords:** growth performance, processed sweet lupin grain, sheep

## Abstract

**Background:**

The experiment evaluated the effect of supplementing sheep fed natural pasture hay withprocessed sweet lupin grain on growth performance and its economic feasibility. The finding revealed that use of steamed lupin shown to improve the nutritivevalue of the grain and sheep performance.

**Methods:**

The experiment was carried out using 24 yearling lambs with initial body weight of 27.53 ± 2.67 kg (mean ± SD) for 126 days (21 days quarantine, 15 days of adaptation and 90 days growth trial followed by 7 days digestibility trial). The experiment was laid out in a randomised complete block design consisting of four treatments and six blocks. Treatments comprised the feeding of natural pasture hay ad libitum + concentrate mix 440 g (T1), natural pasture hay + 440 g/day roasted, coarsely ground sweet lupin grain (T2), natural pasture hay + 440 g/day sweet lupin grain soaked in water for 72 h (T3), natural pasture hay + 440 g/day steamed sweet lupin grain (T4).

**Results:**

There was improvements in total dry matter intake and digestibility coefficients of dry matter, organic matter, crude protein, neutral detergent fibre and acid detergent fibre in sheep supplemented with processed sweet lupin grains compared (T4) by 58.49%, 24.66%, 39.39%, 22.97% and 39.68%, respectively, over the control group. Specifically sheep supplemented with T4 had significantly higher (*p* < 0.001) average daily gain (by 51.04%), feed conversion efficiency (46.34%) and daily weight gain (144.78 g/day) compared to the control treatment, respectively. All processing methods resulted in favourable average daily gain and net return, thus can be employed in feeding systems depending on their availability and relative cost.

**Conclusions:**

Supplementing sheep fed natural pasture hay with 440 g/day steamed sweet lupin grains improved growth performance and fattening economics of Doyogena sheep compared to T2 (roasted sweet lupin grain), T3 (soaked sweet lupin grain) or the control (T1).

## Graphical abstract

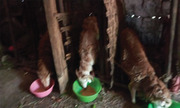

## 1 INTRODUCTION

In Ethiopia, the livestock sub‐sector has a significant contribution to the national income and livelihoods of households (Leta & Mesele, 2014). However, the role of the sector towards the country's economy has not been in line with its potential. This is associated with several complex and inter‐related factors of which inadequate feed is the major one (Osti, 2020). Major feed resources for ruminants in the country include natural pasture, crop stubble, road and riverside pasture, crop residues, and agro‐industrial by‐products (CSA, 2018). Sheep in the highlands depend on communal grazing, fallow lands and crop residues (Kenfo et al., 2018). These feed resources provide insufficient nutrients beyond maintenance requirements leading to low productivity. This situation is aggravated during the dry season when natural pastures are critically deficient in protein and energy content (Kenfo et al., 2018). Thus, supplementation with high nutritive value feed resources is imperative to improve sheep growth performance and productivity in the country.

Sweet lupine (*Lupinus angustifolius L*.) is one of the major crops grown in different soil types. Its growth performance compares better than other lupin species (Tessema, 2017) in all locations. It yields 2.98 t/ha of grain in mid altitudes of Southern Ethiopia (Tessema, 2017) and can be used as an alternative home‐grown protein supplement to mitigate livestock feed shortages in the country (Haile et al., 2017). It has a relatively high crude protein (CP) content of 34.35%, digestible organic matter (DOM) content of 86.28% and a relatively low alkaloid content (Yenesew et al., 2015). The alkaloid content of sweet lupin was found to be 112 mg/kg DM while the white local varieties has high 10,231 mg/kg DM as reported by Likawent et al. (2012). Besides, a study conducted using sweet lupin grain as a supplement with 290 g/head per day on Washera sheep showed that the animals gained 74 g/head per day and 5.1 kg/head in a trial of 69 days (Yeheyis et al., 2012). Supplementation of sweet lupin has potential to improve the efficiency of utilisation of available roughage feed resources (Rodrigues et al., 2017).

It has been reported by Likawent et al. (2012) that the high alkaloid content of local lupin has negative impact on the utilisation of seeds as livestock and human food.

Soaking after roasting, boiling, germination, fermentation and alkaline treatment are practices that have been reported to reduce anti‐nutritional factors in diets (Joray et al., 2007). The practice of processing sweet lupin seed as a supplement for fattening lambs is relatively new in Ethiopia, but it has not been studied. Therefore, this study conducted an on‐farm evaluation to determine promising processing methods of sweet lupin on feed intake, nutrient digestibility and growth performance of Doyogena sheep in Ethiopia.

## 2 MATERIAL AND METHODS

### 2.1 Farmer selection

**FIGURE 1:**
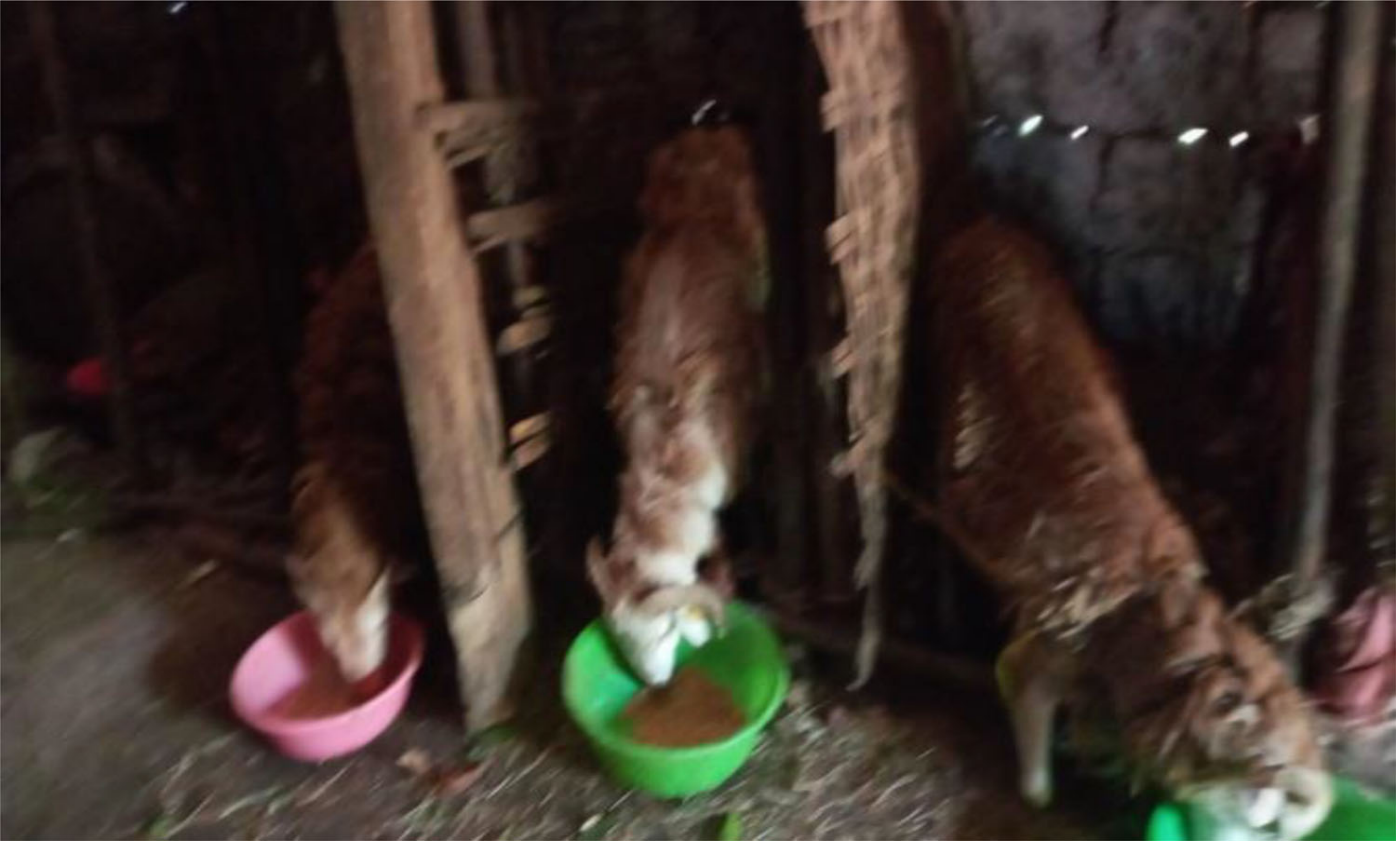
Fattening Doyogena sheep with processed sweet lupin supplementation

The study was undertaken on‐farm. The district comprises of 4 urban and 13 rural PA. Ancha Sadicho, one of the rural PA (peasant association – a lower‐level administrative hierarchy in Ethiopia), was purposively selected based on availability of sufficient sheep populations as well as willingness and interest of farmers to participate. Subsequently, 8 volunteer farmers who owned more than 10 sheep were selected from four different villages within the PA (Figure 1). Selection of the farmers was facilitated by agricultural development agents. Three growing intact male lambs were selected from the flock of each farmer considering uniformity in physical performance, health status and body weight. Before the commencement of the experiment, farmers were trained on experimental procedures.

### 2.2 Experimental animals and their management

Twenty‐four yearling intact male Doyogena sheep (27.53 ± 0.554 kg live weight) were quarantined for 21 days to adapt them to temporary shelter built on‐farm by participant farmers and to observe their health status. During this period, sheep were vaccinated against ovine pasteurellosis, sheep pox, blackleg and anthrax with 1 ml ovine pasteurellosis vaccine, 1 ml sheep pox vaccine and 1/2 ml anthrax vaccine per sheep respectively. Sheep were dewormed against internal parasites (flat worms and round worms), drenched with anthelminthics and sprayed against external parasites (tick and mange) before the beginning of the experiment.

The experimental design was a randomised complete block design. Experimental sheep were blocked into six groups each group with four sheep, based on their initial body weight (BW) and randomly assigned to one of the four dietary treatments. Before commencement of the experiment, initial BW was measured and recorded as an average of three consecutive weightings after overnight fasting. The four villages had equal number of sheep for each treatment. Live weight was measured gravimetrically using a portable spring‐dial hoist scale (Camry, NTB, Camty company, China), with a capacity of 100 kg and precision of 50 g. Animals weight measurements were taken in the morning after overnight fasting to avoid variations due to gut fill. Sheep had free access to clean water and salt throughout the experimental period. Experimental animals were managed jointly by farmers and researchers.

### 2.3 Experimental feed

Experimental feed consisted of a basal diet of natural grass hay, processed sweet lupin grain and commercial concentrate. The concentrate mix was in proportion of 30% noug seed cake, 35% wheat bran, 34% ground maize grain and 1% salts. Natural pasture hay was obtained from the local market and chopped to 5–15 cm prior to offering. Sweet lupin grain was processed by roasting then grinding, soaking in water for 72 h, or steaming. Roasting was done on a flat plate surface (*Mitad*) by continuously mixing and stirring the seeds to ensure uniformity and until several black spots were observed on seeds. The temperature of template surface was 144°C and the average roasting period was 13 min. Roasted grain was, thereafter, ground in an attrition mill. Each kilogram of raw sweet lupin grain was soaked in 2 L of tap water for 72 h. Raw sweet lupin grains were steamed for 30 min.

### 2.4 Experimental design and treatments diets

The experiment was laid down in a randomised complete block design. Experimental animals were blocked into six groups based on their initial body weight (BW), which was determined after two consecutive weights and taking the average weight for each sheep. Then the sheep were and randomly assigned to one of the four dietary treatments using lottery method for treatments as control, natural pasture hay + concentrate mix (T1), natural pasture + 440 g/day roasted, coarsely ground lupin grain (T2), natural pasture + 440 g/day soaked lupin grains (T3) and natural pasture + 440 g/day steamed lupin grain (T4).

### 2.5 Feeding trial

The experimental sheep were adapted to experimental diets for 15 days and actual data collected for 90 days. Treatment feeds were offered to individual sheep twice daily at 10:00 am and 5:00 pm in equal portions. Refusals were collected, sampled and weighed daily per animal and pooled on treatment basis for laboratory analysis. Daily feed intake of each sheep was recorded. Experimental sheep were weighed on the first day of the feeding trial and subsequently at weekly intervals after overnight fasting.

Feed conversion efficiency (FCE) was calculated as follows:

(1) $$\mathrm{Feed}\;\mathrm{conversion}\;\mathrm{efficiency}\;=\frac{\mathrm{Average}\;\mathrm{daily}\;\mathrm{live}\;\mathrm{weight}\;\mathrm{gain}\;\left(g\right)}{\mathrm{Average}\;\mathrm{daily}\;\mathrm{feed}\;\mathrm{intake}\left(g\right)}.\;$$

### 2.6 Digestibility trial

The digestibility trial was conducted after 90 days of the feeding trial. It comprised of 3 days for animals to adapt to carrying the faecal collecting bags followed by 7 days of faeces collection. Faeces were collected and weighed every morning for each animal before offering feed and water. It was kept in airtight plastic containers and stored at –20°C in a deep freezer refrigerator up to completion of the digestibility trial. Feed offered, and refusals were collected, weighed and recorded every morning. At the end of the digestibility trial, faecal samples were pooled for each sheep, thawed, thoroughly mixed and 20% subsample and dried at 60°C for 48 h. Faecal samples for determination of dry matter digestibility were air‐dried and kept until oven drying at 105°C. Partially dried faeces were ground to pass through a 1 mm sieve and stored until laboratory analysis.

### 2.7 Chemical analysis

Representative samples of feed offered, refusals, and faecal samples were analysed at the ILRI Animal Nutrition Laboratory in Addis Ababa. Samples of feed offered, refusals and faeces were dried at 60°C for 48 h in a forced draft oven and ground to pass through 1 mm sieve using a Wiley mill, packed into paper bags and stored pending further laboratory works. They were analysed for dry matter (DM), ash, crude protein (CP), neutral detergent fibre (NDF), acid detergent fibre (ADF), acid detergent lignin (ADL), in vitro organic matter digestibility (IVOMD) and metabolisable energy (ME) using the sweet lupin near‐infrared reflectance spectroscopy (NIRS) prediction equation (Glencross et al., 2008). Prior to scanning using NIRS, ground samples were dried overnight at 60°C to standardise moisture conditions. Crude protein (%) of feed samples was determined by multiplying the N content of the samples with the conversion factor of 6.25. The metabolisable energy of feeds offered was estimated from digestible energy (DE) and IVOMD using regression and summation equations developed by NRC (2001): Digestible energy was obtained using the formula:

(2) $$\begin{array}{c} \mathrm{DE}=\left(0.01\times \left(\mathrm{OM}/100\right)\times \left(\mathrm{IVOMD}+12.9\right)\times 4.4\right)-0.3,\\ \mathrm{ME}\left(\mathrm{mcal}/\mathrm{kg}\right)=0.82\times \mathrm{DE}\mathrm{was}\mathrm{calculated}\mathrm{and}\mathrm{converted}\mathrm{to}\mathrm{kilogram}\\ \mathrm{ME}\left(\mathrm{MJ}/\mathrm{kg}\right)=4.184\times \mathrm{ME}\left(\mathrm{mcal}/\mathrm{kg}\right). \end{array}$$

where DE is digestible energy; IVOMD is in vitro organic matter digestibility; ME is metabolisable energy; MJ is megajoule; mcal is megacalorie; kg is kilogram.

### 2.8 Partial budget analysis

Partial budget analysis was used to determine the profitability of the feeding regime on the experimental sheep. The purchase price of experimental feed was recorded and also market price of experimental sheep at the beginning and at the end of the experiment was assessed in the local animal market and estimated by experienced sheep dealers in the district. The partial budget analysis was calculated from the variable costs and benefits. At the same time, three experienced local sheep dealers were selected purposively for estimating the selling price of each experimental sheep before and after supplementation and the average of those three‐estimation prices was taken. Then, the variable costs were calculated from supplementary feeds, which are provided for each experimental sheep treatment costs. The total returns (TR) was determined by calculating the difference between the estimated selling prices and purchasing price of experimental sheep and the cost of the supplemented feed. Net return (NR) was calculated as NR = TR – TVC. The change in net return (ΔNR) was calculated as the difference between a change in total return (ΔTR) and the change in total variable costs (ΔTVC) (supplemented feed), ΔNR = ΔTR – ΔTVC. The marginal rate of return (MRR) measures the increase in net income (∆NR) associated with each additional unit of expenditure (∆TVC) was calculated as MRR = (∆NR/∆TVC) × 100.

To determine the economic return of the feeding trial, partial budget analysis was calculated according to Upton (1979), as follows:

Total return (TR) = selling price − purchasing price of the sheep,
Net return (NR) = TR − total variable cost (TVC),
Change in net return (NR) = NR of supplemented treatment − NR of controlled treatment,
Change in total variable cost (TVC)  = TVC of supplemented treatment − TVC of controlled treatment.
To measure the increase in net return associated with each additional unit of expenditure, the marginal rate of return (MRR) was calculated as MRR = NR/TVC.

### 2.9 Statistical analysis

All data from the trial experiments, on feed intake, feed quality (nutritional content of feed) and live weight gain, FCE, and others were summarised and managed with MS‐Excel (2010) and then subjected to analysis of variance (ANOVA) in a randomised complete block design using the general linear model procedure of SAS (2002) version 9.00. Individual differences between means had been tested using Duncan's multiple range test.

## 3 RESULTS

### 3.1 Chemical composition of experimental feeds

The result of the chemical composition of the experimental feeds is presented in Table 1. There was higher amount of OM, CP, ME and IVOMD in the conc. mix, RCGSLG, SSLG and StSLG feed components as compared to hay feed, the NDF, ADF and ADL as well as hemicelluloses components of hay were higher than other feed components as shown in the table.

**TABLE 1:** Chemical composition of experimental feed

	Treatment feed offered (%)				
Nutrient parameter	Hay	Conc. mix	RCGSLG	SSLG	StSLG
DM (%)	91.2	92.3	93.4	93.3	93.1
Ash (%DM)	14.3	5.6	4.4	4.5	3.9
OM (%DM)	85.7	94.4	95.6	95.5	96.1
CP (%DM)	6.8	17.6	36.2	39.3	28.2
NDF (%DM)	66.7	32.1	30.2	30.3	38.7
ADF (%DM)	40.9	16.3	16.8	14.7	25.3
ADL (%DM)	6.7	3.9	2.0	1.1	1.6
ME (MJ/kg)	6.9	9	11.3	11.3	10.2
IVOMD (%)	47.7	62.8	80.6	81.7	72.8
Hemicellulose	25.8	15.8	13.4	15.6	13.4

DM, dry matter; OM, organic matter; CP, crude protein; NDF, neutral detergent fibre; ADF, acid detergent fibre; ADL, acid detergent lignin; con. mix, concentrate mixture (30%, 35%, 35% and 1% noug seed cake, coarsely ground maize grain, wheat bran and salt, respectively); RCGSLG, roasted and coarsely ground sweet lupin grain; SSLG, soaked sweet lupin grain; StSLG, steamed sweet lupin grain; ME, metabolisable energy; IVOMD, in vitro organic matter digestibility.

### 3.2 Dry matter and nutrient intake

The DMI of hay in T4 was significantly higher than T1 (Table 2). The DMI of lupin grains was significantly higher (*p* < 0.05) than the concentrate mix. The total DMI and OMI in T4 was significantly higher (*p* < 0.05) than T1, which has shown a 2.66% increment. NDF intake of T4 was significantly higher (*p* < 0.05) than the control. Sheep in T2 and T3 had no significance (*p* > 0.05) difference in terms of total DMI, OMI and NDF except CP and ADF intakes where these nutrients found to be significant between these treatments. Sheep in the T3 and T4 had significantly less (*p* < 0.05) ADF intake compared to sheep in the T1.

**TABLE 2:** Dry matter and nutrient intake of Doyogena lambs fed natural pasture hay basal feed, supplemented with processed sweet lupin grain

	Treatments					
Dry matter intake	T1	T2	T3	T4	SEM	*p* Value
Hay DMI	378.79^b^	385.14^ab^	382.21^ab^	395.8^a^	2.81	0.1590
Supplement DMI	405.23^d^	411.04^a^	410.56^b^	409.61^c^	0.41	<0.0001
Total DMI	784.76^b^	796.18^ab^	792.77^ab^	805.46^a^	2.92	0.0808
DMI (g/kg W)	53.60	50.46	51.43	51.53	1.84	ns
Nutrient intake (g/day)						
Total ASH	84.03^b^	79.74^c^	80.16^c^	99.46^a^	1.71	<0.0001
Total OM	770.83^b^	782.51^ab^	779.32^ab^	794.77^a^	2.94	0.0233
Total CP	105.68^d^	187.68^b^	200.97^a^	153.59^c^	7.67	<0.0001
Total NDF	418.24^b^	414.52^b^	412.82^b^	459.75^a^	4.4	<0.0001
Total ADF	241.57^bc^	246.18^b^	236.07^c^	288.82^a^	4.49	<0.0001

^a–d^Means with different superscript within a row differ (*p* < 0.05) and (*p* < 0.01).

BW, body weight of live animal; DMI, dry matter intake; NS, non‐signifant; OMI, organic matter intake; NDF, neutral detergent fibre intake; ADF, acid detergent fibre; ADL, acid detergent lignin; CP, crude protein intake; SEM, standard error mean; SL, significant level; T1, concentrate mixture; T2, roasted and coarsely ground sweet lupin grain; T3, soaked sweet lupin grain; T4, steamed sweet lupin grain; ns, non‐significant.

### 3.3 Dry matter and nutrients digestibility

The DM, OM, CP, NDF and ADF digestibility of the lupin groups was significantly higher (*p* < 0.05) than the control as indicated in Table 3. There was an improvement of 58.49%, 24.66%, 39.39%, 22.97% and 39.68% increment in T4 over T1 (the control group). Regarding the CP digestibility, there was no significant difference between T2 and T4 groups. However, there was significant (*p* < 0.05) difference between T2 and T3 in terms of all nutrients’ digestibility.

**TABLE 3:** Apparent nutrient digestibility coefficients of treatment feeds in Doyogena sheep fed on hay and supplemented with processed sweet lupin grain

	Treatments					
	T1	T2	T3	T4	SEM	*p* Value
DM	0.53^d^	0.78^b^	0.69^c^	0.84^a^	0.029	<0.0001
OM	0.73^d^	0.87^b^	0.80^c^	0.91^a^	0.028	<0.0001
CP	0.66^c^	0.92^a^	0.84^b^	0.92^a^	0.063	<0.0001
NDF	0.74^d^	0.86^b^	0.79^c^	0.91^a^	0.039	<0.0001
ADF	0.63^d^	0.80^b^	0.72^c^	0.88^a^	0.063	<0.0001

^a–d^Means within a row with different superscript differ (*p* < 0.01).

SEM, standard error of means; DM, dry matter; CP, crude protein; OM, organic matter; NDF, neutral detergent fibre; ADF, acid detergent fibre; SL, significance level; T1, concentrate mixture; T2, roasted and coarsely ground sweet lupin grain; T3, soaked sweet lupin grain; T4, steamed sweet lupin grain.

### 3.4 Bodyweight change and feed conversion efficiency

Of the parameters measured related to body weight, the average daily gain and FCE of T4 were significantly higher (*p* < 0.05) than that of T1 (Table 4). These parameters showed 51.04% and 46.34% increments, respectively, in T4 over the control. However, there was no significant difference between T2 and T3 in the above parameters.

**TABLE 4:** Bodyweight parameters and feed conversation efficiency of Doyogena sheep fed on natural pasture hay and supplemented with processed sweet lupin grain

	Treatment					
Parameters	T1	T2	T3	T4	SEM	*p* Value
IBW (kg)	27.4	28.16	28.23	26.38	0.55	0.6283
FBW (kg)	36.08	39.88	38.83	39.41	0.86	0.4234
ADG (Kg)	0.096^b^	0.130^ab^	0.117^ab^	0.145^a^	0.006	0.0467
BWC (kg)	8.71^b^	11.72^ab^	10.56^ab^	13.03^a^	0.58	0.0467
FCE	0.123^b^	0.163^ab^	0.148^ab^	0.180^a^	0.008	0.0822

^a–b^Means within rows with different superscripts differ (*p* < 0.01).

IBW, initial body weight; FBW, final body weight; BWC, body weight change; ADG, average daily weight gain; FCR, feed conversion efficiency; SEM, standard error of means; NS, non‐significant; SL, significance level; kg, kilogram; T1, concentrate mixture; T2, roasted and coarsely ground sweet lupin grain; T3, soaked sweet lupin grain; T4, steamed sweet lupin grain.

### 3.5 Partial budget analysis

Partial budget analysis of Doyogena sheep fed on hay basal diet and supplemented with different forms of processed lupin grain is given in Table 5. All lupin supplemented groups tended to have higher net returns over the control feed sheep groups as shown in the table.

**TABLE 5:** Partial budget and marginal rate of return analysis for Doyogena sheep supplemented with processed sweet lupin grain on hay‐based feeding

	Treatments			
Parameters	T1	T2	T3	T4
Purchase price of sheep (ETB/head)	2025.0	1952.8	2147.2	2175.0
Total hay consumed (kg/head)	34.9	35.5	35.2	36.5
Feed cost for hay (ETB/head)	102.4	104.1	103.3	107.0
Total concentrate consumed (kg/head)	50.5	50.5	50.5	50.5
Cost for concentrates (ETB/head)	480.0	–	–	–
Cost of feed (ETB/kg)	–	333.0	333.0	333.0
Total feed cost (ETB/head)	582.4	437.1	436.5	440.0
Gross income (ETB/head)	4413.9	5816.7	6127.8	6383.3
Total return (ETB/head)	1806.5	3426.8	3544.1	3768.4
Total variable cost (ETB/head)	1120.0	2133.3	2458.3	2458.3
Net return (ETB/head)	686.5	1293.5	1085.7	1310.0
∆NR	–	1147.01	45.45	223.57
∆TVC	–	327.96	71.27	4.19
MRR (%)	–	3.497	0.637	53.357

ETB, Ethiopian Birr; ∆NI, change in net income; ∆TVC, change in total variable cost; MRR, marginal rate of return; T1, concentrate mixture; T2, roasted and coarsely ground sweet lupin grain; T3, soaked sweet lupin grain; T4, steamed sweet lupin grain.

## 4 DISCUSSION

It had been stated that CP content ranging between 7% and 7.5% is required to satisfy ruminant microbial demands for nitrogen that would provide sufficient CP for the maintenance requirement of the animals (Van Soest, 1982). The CP content of natural pasture hay in the current finding was lower than the previously reported (Ali et al., 2019) from natural pasture hay but higher than 3.88%, 5.28% and 6.45% reported by Mekuriaw and Asmare (2018), Dejene (2017) and Demoze (2020), respectively, in different parts of Ethiopia. Nevertheless, the observed CP content of natural grass hay used in this on‐farm feeding trial was found to be slightly below the maintenance requirements of ruminant animals (sheep). The current CP content in the natural pasture or hay indicates that it requires supplementation with better quality feed to meet not only maintenance requirements but also productivity of animals. On the other hand, the CP content of roasted and coarsely ground sweet lupin grain is high, which enable it to be used to supplement sheep fed poor basal diet. Thus, the use of lupin in the current study is one of the strategies that can mitigate shortage of protein for ruminants in developing countries.

### 4.1 Dry matter and nutrient intake

The total dry matter and nutrient intakes of sheep in the current study improved by supplementation of processed lupin. This is what has been supposed to be obtained in such kinds of studies as the main target of use of protein feeds is to improve the intake of dry matter and nutrients intakes. The dry matter intake normalised for live weight of sheep was not negatively affected by the dietary treatment. That means feeding lupin grains did not affect the palatability of sheep, instead it positively influenced the overall performance of animals. This finding is in agreement with Yilkal et al. (2014) and Yeheyis et al. (2012) reported for supplementation with different forms of processed lupin (*Lupinus albus*) grain in hay‐based feeding of Washera sheep. Moreover, in the current experiment, the different legume and processing methods did not affect the performance of lambs’ performances (Lestingi et al., 2019). On the other hands, Tefera et al. (2015) also reported that supplementation of processed sweet lupin grain improved body weight changes and carcass yield of lambs in Ethiopia. The inconsistency of findings might be related to the types of basal diet used and processing methods of lupin grains in respective studies.

### 4.2 Dry matter and nutrients digestibility

The findings showed an increment in the digestibility of nutrients particularly in T4. This improvement in nutrient digestibility in T4 means that steaming lupin grains decreased the anti‐nutritional factors, which otherwise might have influence digestibility of nutrients in the grain. The effect of processing and its importance in decreasing anti‐nutritional values has been also reported by other researchers earlier (Yilkal et al., 2014; Likawent et al., 2012) who reported that the digestibility of nutrients of lupin grains improves by several physical treatments. Animals in T2 and T3 had shown intermediate results in both final body weight and FCE. The improvement in weight gain and FCE in T4 observed in the current result might be due to the increase in nutrient supply as a result of the increase in digestibility. This is in agreement with Yilkal et al. (2014) and Yeheyis et al. (2012) studies on lupin grains. The improvement in the total net return suggests that the use of steamed lupin grain is a reliable option to replace the expensive commercial concentrate in meat production in Ethiopian rural areas. Steaming grains is high‐demanding process for water and energy, which are limited in the rural areas of Ethiopia. To expand the practice of use of steamed lupin grain, optimising this process (decreasing the duration, temperature and amount of water for steaming) should be identified before large‐scale promotion of steaming as a process to improve the nutritive value of lupin grains. However, Arfaoui et al. (2020) stated that supplementation of sweet lupin for Barbarin Lamb had no significant effect on total dry matter and water intakes, average daily gain, diet digestibility and microbial synthesis. The inconsistency of findings might be related to genetic variation of sheep, environmental conditions of the animals and management of animals.

### 4.3 Partial budget analysis

The marginal rate of return or ratio for supplemented sheep in T2 and T4 was 3.497 and 53.357ETB (1.10 USD), respectively. The result achieved in the present study was higher than 1.22 ETB (0.025 USD) reported by Amanie et al. (2017) but lower than sheep supplemented with soaked sweet lupin grain (T3). This might be due to variations in the purchasing price of sheep, current market situation and selling price of sheep, variations in sheep breeds used and differences in basal diets and supplements used in different experiments.

## 5 CONCLUSION

From the current finding, it is understood that steamed lupin grains are more suitable and economical as compared to other types of lupin processing and presumed to be good replacement for commercial concentrate mixed to fattening sheep. The supplements used in this study showed increment in an average daily gain and net return and thus can be used in feeding systems depending on their availability and relative costs. The use of steamed lupin improves the nutritive value of the grain and sheep performance in the current study. For wider use of steamed lupin in the area, the simplification of the steaming process of lupin grains should be studied prior to the final recommendation of this treatment.

## CONFLICT OF INTEREST

The authors declare that they have no conflict of interest.

## ETHICS STATEMENT

The experiment was conducted according to the experimental animal management of Ethiopian research system.

## AUTHOR CONTRIBUTIONS

All the authors contributed in research conceptualisation, experimental design, data collection and data curation, analysis and final draft manuscript writing.

## CONSENT FOR PUBLICATION (INCLUDE APPROPRIATE STATEMENTS)

All authors agreed on the publication of this paper and assigned corresponding author responsible in charge for correspondence during manuscript publishing.

### PEER REVIEW

The peer review history for this article is available at https://publons.com/publon/10.1002/vms3.883.

## Data Availability

Data are available from the corresponding author upon request.
